# Supplementary material for: Development of a multiplex real-time PCR assay for the simultaneous detection of four bacterial pathogens causing pneumonia
Source: PLoS One. 2021 Jun 17;16(6):e0253402. doi: 10.1371/journal.pone.0253402 (PMC8211157; doi:10.1371/journal.pone.0253402)
Supplement: S1 Table — The RB4 mRT-PCR assays detected an additional 19 bacterial infections from reference-positive assays. (PDF) [file pone.0253402.s001.pdf]

Sample ID	Discordance type	Reference assays	RB4 mRT-PCR
A	Single infection	*K. pneumoniae*	*K. pneumoniae, P. aeruginosa*
B		*K. pneumoniae*	*K. pneumoniae., P. aeruginosa*
C		*K. pneumoniae*	*K. pneumoniae, P. aeruginosa*
D		*P. aeruginosa*	*P. aeruginosa, M. catarrhalis*
E		*P. aeruginosa*	*K. pneumoniae, P. aeruginosa*
F		*P. aeruginosa*	*K. pneumoniae, P. aeruginosa*
G		*S. aureus*	*K. pneumoniae, S. aureus*
H		*S. aureus*	*P. aeruginosa, S. aureus*
I		*M. catarrhalis*	*K. pneumoniae, M. catarrhalis*
J		*M. catarrhalis*	*P. aeruginosa, M. catarrhalis*
K		*K. pneumoniae, P. aeruginosa*	*K. pneumoniae, P. aeruginosa,, S. aureus*
L		*K. pneumoniae, S. aureus*	*K. pneumoniae, P. aeruginosa, S. aureus*
M		*K. pneumoniae, S. aureus*	*K. pneumoniae, P. aeruginosa, S. aureus*
N		*K. pneumoniae, S. aureus*	*K. pneumoniae, P. aeruginosa, S. aureus*
O		*P. aeruginosa, S. aureus*	*K. pneumoniae, P. aeruginosa., S. aureus*
P	mixed infections	*K. pneumoniae*	*K. pneumoniae, P. aeruginosa, S. aureus*
Q		*P. aeruginosa*	*K. pneumoniae, P. aeruginosa, S. aureus*
R		*S. aureus*	*K. pneumoniae, P. aeruginosa, S. aureus*
S		*S. aureus*	*K. pneumoniae P. aeruginosa, S. aureus*
