# Supplementary material for: Development of a multiplex real-time PCR assay for the simultaneous detection of four bacterial pathogens causing pneumonia
Source: PLoS One. 2021 Jun 17;16(6):e0253402. doi: 10.1371/journal.pone.0253402 (PMC8211157; doi:10.1371/journal.pone.0253402)
Supplement: Attachment — Submitted filename: Response_to_Reviewers.docx [file pone.0253402.s002.docx]

**Reviewers' comments:**

Reviewer's Responses to Questions

**Comments to the Author**

1. Is the manuscript technically sound, and do the data support the conclusions?

The manuscript must describe a technically sound piece of scientific research with data that supports the conclusions. Experiments must have been conducted rigorously, with appropriate controls, replication, and sample sizes. The conclusions must be drawn appropriately based on the data presented.

Reviewer #1: Yes

Reviewer #2: Yes

2. Has the statistical analysis been performed appropriately and rigorously?

Reviewer #1: Yes

Reviewer #2: Yes

3. Have the authors made all data underlying the findings in their manuscript fully available?

The [PLOS Data policy](http://www.plosone.org/static/policies.action#sharing) requires authors to make all data underlying the findings described in their manuscript fully available without restriction, with rare exception (please refer to the Data Availability Statement in the manuscript PDF file). The data should be provided as part of the manuscript or its supporting information, or deposited to a public repository. For example, in addition to summary statistics, the data points behind means, medians and variance measures should be available. If there are restrictions on publicly sharing data—e.g. participant privacy or use of data from a third party—those must be specified.

Reviewer #1: Yes

Reviewer #2: Yes

4. Is the manuscript presented in an intelligible fashion and written in standard English?

PLOS ONE does not copyedit accepted manuscripts, so the language in submitted articles must be clear, correct, and unambiguous. Any typographical or grammatical errors should be corrected at revision, so please note any specific errors here.

Reviewer #1: Yes

Reviewer #2: Yes

5. Review Comments to the Author

Reviewer #1:

The manuscript was well designed and has technical rigor. The study question was answered. The proposed technique may help the diagnosis of pneumonia and will prevent the indiscriminate use of antibiotics.

Reviewer #2:

The new mPCRs are promising tools to help clinicians to make clinical decisions in antibiotic treatments. The authors presented a promising array which could be useful in a clinical setting. It would be extremely useful information (and significantly strengthens the utility of the assay) to know if the assay is able to differentiate antibiotic resistant strain of the different types of bacteria.

1) Any analysis for hairpins etc for the PCR primers?
A: We checked the secondary structures of oligomers (primers and probes) using the GeneRunner software. We tested all 15 oligomers used in this study, and five showed no secondary structures. However, the predicted secondary structures did not significantly affect our PCR-based methods using dual-priming oligonucleotides (DPO) because the 30-mer oligo alone, unlike the 50-mer oligo (#1, 2), was not stable enough to form secondary structures. The GC content (<65 %) and the avoidance of repetitive sequences met the oligo design criteria (#3).

- References –

1. Chun JY, Kim KJ, Hwang IT, Kim YJ, Lee DH, Lee IK, et al. Dual priming oligonucleotide system for the multiplex detection of respiratory viruses and SNP genotyping of CYP2C19 gene. Nucleic Acids Res. 2007;35(6):e40. Epub 2007/02/09. doi: 10.1093/nar/gkm051. PubMed PMID: 17287288; PubMed Central PMCID: PMCPMC1874606.

2. Fredman D, Jobs M, Strömqvist L, Brookes AJ. DFold: PCR design that minimizes secondary structure and optimizes downstream genotyping applications. Hum Mutat. 2004;24(1):1-8. doi: 10.1002/humu.20066. PMID: 15221783.

3. Riet J, Ramos LRV, Lewis RV, Marins LF. Improving the PCR protocol to amplify a repetitive DNA sequence. Genet Mol Res. 2017;16(3). doi:10.4238/gmr16039796.

2) Have you investigated your assay against antibiotic resistant strains?

A: Yes, RB4 mRT-PCR assays worked for antibiotic-resistant pathogens. Fifty-eight samples used in this study were tested for antibiotic resistance as a part of the minimal inhibitory concentration (MIC) test procedure.

**<Data table>**

| **Pathogen** | **Total samples (*N*)** | **MIC test (*%*)** | **Antibiotic-resistant (*N*)** |
| --- | --- | --- | --- |
| *K. pneumoniae* | 66 | 20 (30.3%) | 20 |
| *P. aeruginosa* | 70 | 29 (41.4%) | 27 |
| *S. aureus* | 55 | 10 (18.2%) | 10 |
| *M. catarrhalis* | 18 | 0 (0%) | 0 |

Data are representative of the total samples, MIC test, and antibiotic resistance for target pathogens in 58 samples.

3) Have you investigated the antibiotic susceptibility data against the results from the assay?

A: Yes. *K. pneumoniae*, *P. aeruginosa*, and *S. aureus* were analyzed by MIC tests for resistance to 37 antibiotics and classified into three categories: antibiotic-resistant, antibiotic-intermediate, or antibiotic-susceptible. We analyzed the association of antibiotic resistance with extended-spectrum beta-lactamase (*ESBL*) gene types in *K. pneumoniae* strains. We screened 17, 17, and 16 antibiotics for *K. pneumoniae,* *P. aeruginosa,* and *S. aureus*, respectively.

**<Data table>**

| **Antibiotic** | ***K. pneumoniae*** | ***P. aeruginosa*** | ***S. aureus*** |
| --- | --- | --- | --- |
| Amikacin | S | S | N/A |
| Amoxicillin/Clavulanic acid | R | N/A | N/A |
| Ampicillin | R | N/A | N/A |
| Ampicillin/Sulbactam | N/A | R | N/A |
| Aztreonam | R | S | N/A |
| Cefazolin | R | N/A | N/A |
| Cefepime | R | S | N/A |
| Cefotaxime | R | R | N/A |
| Cefoxitin | S | N/A | N/A |
| Ceftazidime | R | S | N/A |
| Ciprofloxacin | R | S | R |
| Clindamycin | N/A | N/A | R |
| Colistin | N/A | S | N/A |
| Ertapenem | S | N/A | N/A |
| Erythromycin | N/A | N/A | R |
| Esbl | + | N/A | N/A |
| Fusidic acid | N/A | N/A | S |
| Gentamicin | S | S | S |
| Imipenem | S | S | N/A |
| Levofloxacin | N/A | N/A | N/A |
| Linezolid | N/A | N/A | S |
| Meropenem | N/A | S | N/A |
| Mikacin | N/A | N/A | N/A |
| Minocycline | N/A | R | N/A |
| Oxacillin | N/A | N/A | R |
| Penicillin G | N/A | N/A | R |
| Piperacillin | N/A | I | N/A |
| Piperacillin/Tazobactam | R | I | N/A |
| Quinupristin/Dalfopristin | N/A | N/A | S |
| RFP (Rifampicin) | N/A | N/A | S |
| Teicoplanin | N/A | N/A | S |
| Telithromycin | N/A | N/A | R |
| Tetracycline | N/A | N/A | S |
| Ticarcillin/Clavulanic acid | N/A | S | N/A |
| Tigecycline | S | R | S |
| Tobramycin | N/A | N/A | N/A |
| Trimethoprim/Sulfamethoxazole | S | R | S |
| Vancomycin | N/A | N/A | S |

Data are representative of antibiotics for target pathogens in 58 samples. Abbreviations: R; resistant; I, intermediate; S, susceptible; N/A; not available.

We hope you are satisfied with our answer.

Thank you very much.

6. PLOS authors have the option to publish the peer review history of their article ([what does this mean?](https://journals.plos.org/plosone/s/editorial-and-peer-review-process#loc-peer-review-history)). If published, this will include your full peer review and any attached files.


If you choose “no”, your identity will remain anonymous but your review may still be made public.


**Do you want your identity to be public for this peer review?** For information about this choice, including consent withdrawal, please see our [Privacy Policy](https://www.plos.org/privacy-policy).

Reviewer #1: No

Reviewer #2: No

*In compliance with data protection regulations, you may request that we remove your personal registration details at any time.* [*(Remove my information/details)*](https://www.editorialmanager.com/pone/login.asp?a=r)*. Please contact the publication office if you have any questions.*
